# Impact of Seasonality on Recruitment, Retention, Adherence, and Outcomes in a Web-Based Smoking Cessation Intervention: Randomized Controlled Trial

**DOI:** 10.2196/jmir.2880

**Published:** 2013-11-07

**Authors:** Amanda L Graham, Sarah Cha, Nathan K Cobb, Ye Fang, Raymond S Niaura, Aaron Mushro

**Affiliations:** ^1^Schroeder Institute for Tobacco Research and Policy StudiesLegacyWashington, DCUnited States; ^2^Department of Oncology, Georgetown University Medical Center / Cancer Prevention and Control, Lombardi Comprehensive Cancer CenterWashington, DCUnited States; ^3^Department of Health, Behavior and SocietyThe Johns Hopkins Bloomberg School of Public HealthBaltimore, MDUnited States; ^4^Department of Pulmonary, Critical Care, and Sleep MedicineGeorgetown University Medical CenterWashington, DCUnited States; ^5^MeYou HealthBoston, MAUnited States; ^6^Marketing DepartmentLegacyWashington, DCUnited States

**Keywords:** seasonal variation, smoking cessation, Internet, research subject recruitment

## Abstract

**Background:**

Seasonal variations in smoking and quitting behaviors have been documented, with many smokers seeking cessation assistance around the start of the New Year. What remains unknown is whether smokers who are recruited to cessation treatment trials during the New Year are as motivated to quit, or as likely to enroll in a research trial, adhere to a research protocol, and benefit from a cessation intervention compared to those who are recruited during other times of the year.

**Objective:**

The objective of this study was to determine whether smokers recruited during the New Year period differ on measures of motivation and desire to quit, recruitment and retention rates, website utilization rates, and short-term cessation outcomes compared to smokers recruited at other times.

**Methods:**

Participants were current smokers who had registered on a free Web-based cessation program (BecomeAnEX.org) and were invited to participate in a clinical trial. The New Year period was defined according to a clear peak and drop in the proportion of visitors who registered on the site, spanning a 15-day period from December 26, 2012 to January 9, 2013. Two other 15-day recruitment periods during summer (July 18, 2012 to August 1, 2012) and fall (November 7, 2012 to November 21, 2012) were selected for comparison. Data were examined from 3 sources: (1) a Web-based clinical trials management system that automated the recruitment and enrollment process, (2) self-report assessments at baseline and 3 months postrandomization, and (3) online tracking software that recorded website utilization during the first 3 months of the trial.

**Results:**

Visitors to BecomeAnEX during the New Year period were more likely to register on the site than smokers who visited during summer or fall (conversion rates: 7.4%, 4.6%, 4.9%, respectively; *P*<.001), but there were no differences in rates of study acceptance, consent, randomization, 3-month follow-up survey completion, or cessation between the 3 periods. New Year participants were older, more educated, more likely to be employed full time, and more likely to have a relationship partner compared with participants recruited at other times during the year, but did not differ on measures of motivation and desire to quit.

**Conclusions:**

Smokers visiting a Web-based cessation program during the New Year period were more likely to register for treatment and differ on several demographic variables, but showed similar patterns of treatment engagement, retention, follow-up, and short-term cessation outcomes compared with participants who visited the site during other periods of the year. These results allay scientific concerns about recruiting participants during this time frame and are reassuring for researchers conducting Web-based cessation trials.

**Trial Registration:**

ClinicalTrials.gov ID: NCT01544153; http://clinicaltrials.gov/ct2/show/NCT01544153 (Archived by WebCite at http://www.webcitation.org/6KjhmAS9u).

## Introduction

**Figure 1:**
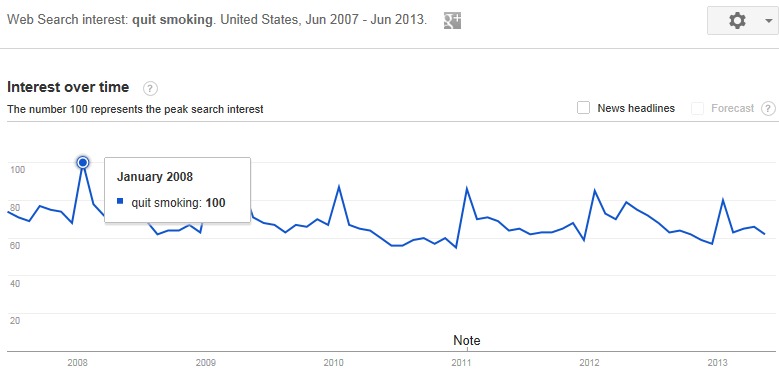
Use of search term “quit smoking” in Google search engine queries relative to the total number of Google searches between June 2007 and June 2013 in the United States as reported in Google Trends.

Seasonal variations across a number of smoking and quitting behaviors have been documented. Most smokers express a desire to quit [1] and many make a quit attempt around the start of the New Year [2-6]. Reports have shown that sales of cigarettes are at their lowest during January and February [7,8] and sales of nicotine replacement therapies are at their highest January through March [9]. Seasonal variations in motivational stage of change among callers to a state quitline have also been documented [10] with callers in December and January being more likely to have recently quit than callers during other months. Internet search queries also provide evidence of the seasonal variations in smoking cessation, with clear peaks observed in the use of “quit smoking” as a search term at the beginning of each calendar year. Figure 1 shows the relative use of the search term “quit smoking” in Google search engine queries over 6 years in the United States as reported by Google Trends [11], the public database of Google queries.

A greater number of smokers quitting around the New Year may mean a greater pool of potential research participants for smoking cessation trials. However, the effects of seasonality on research recruitment and retention have not been documented. Specifically, it is unknown whether smokers who are invited to cessation treatment trials during this period of time are as likely to enroll, to adhere to a research protocol, and to benefit from a cessation intervention. Smokers who elect to quit around the New Year may differ from those who quit during other times of the year on factors such as motivation, desire, confidence, or other factors that relate to trial participation, engagement, and cessation outcomes.

These are important questions to address from both a pragmatic and a scientific standpoint. From a pragmatic standpoint, conducting trial recruitment during the New Year holiday may have staffing and cost considerations for all aspects of a trial. Research staff may be needed to field study inquiries, conduct eligibility screening, administer assessments, and manage study communications; intervention staff may be required to orient new participants to the trial and begin intervention delivery. Given the potential for higher recruitment volume during this period, staffing increases may be required. From a scientific standpoint, if participants enrolled during this time are less likely to adhere to research protocol (ie, lower rates of intervention adherence, lower retention at follow-up) because of a more transient commitment to quitting, this could have important implications for treatment trials. Lower retention rates (ie, higher loss to follow-up) would result in a higher proportion of participants counted as smokers in intention-to-treat analyses, which may artificially deflate overall abstinence rates and perceived effectiveness of an intervention. Lower rates of intervention adherence among participants recruited during the New Year could influence metrics of intervention feasibility and receptivity as well as cessation outcomes.

We sought to examine these questions about the impact of seasonality on smoking cessation treatment trials in the context of an ongoing randomized trial of a Web-based cessation intervention. We extracted a subset of participants recruited during a 15-day window that spanned the 2013 New Year and compared them to participants recruited during 2 other 15-day periods in 2012 on recruitment and retention rates, baseline characteristics, website utilization, and cessation outcomes. Our a priori hypotheses were that New Year participants would differ on baseline measures of motivation and desire to quit consistent with a more transient commitment to quitting, and would have lower recruitment and retention rates, lower website utilization rates, and poorer cessation outcomes compared to participants enrolled during other periods.

## Methods

### Study Overview

The full study protocol has been published elsewhere [12]. Briefly, this is an ongoing Web-based randomized trial to compare the efficacy of an interactive, evidence-based, smoking cessation website alone and in conjunction with (1) a theory-driven, empirically informed social network intervention designed to integrate participants into an online community, and (2) access to a free supply of nicotine replacement therapy (NRT) products. The study uses a 2×2 factorial design to compare the following treatment conditions: (1) website, (2) website+social network intervention, (3) website+NRT, and (4) website+social network intervention+NRT. A total of 4000 participants will be randomized by the end of the study. Follow-up assessments are administered at 3- and 9-months postrandomization; 30-day point prevalence abstinence is the primary outcome of the parent trial. Study eligibility criteria are current smoking, age 18 years or older, and US residence. Exclusion criteria are contraindications to NRT (pregnant or breastfeeding, recent cardiac problems, current NRT use). Randomization is stratified by gender and baseline motivation.

### Recruitment

The study is conducted within BecomeAnEX.org, a free, publicly available, evidence-based intervention developed in accordance with the 2008 US Department of Health and Human Service’s Clinical Practice Guidelines [13]. The site was developed by Legacy, a nonprofit organization that develops smoking prevention and cessation programs, in collaboration with the Mayo Clinic Nicotine Dependence Center [14]. A national multichannel media campaign that included television, radio, and outdoor and online advertising was launched in 2008 to promote the website [15]. The present implementation of this campaign relies on various forms of online advertising, including social media, search engine marketing, and large targeted ad networks for display advertising. Search engine advertising targets keywords related to BecomeAnEX (eg, quit smoking, stop smoking) and display advertising targets males and females aged between 25 and 54 years.

Participants are recruited to the trial immediately following registration on BecomeAnEX. The entire recruitment and enrollment process is automated using a Web-based clinical trials management system. Individuals that indicate current smoking (every day/some days) during registration are invited to the study. Interested individuals complete online eligibility screening; eligible individuals provide online informed consent and contact information, including an email address that is used to send a link to the online baseline assessment. Participants are randomized to treatment upon completion of the baseline survey. No incentive is provided for enrollment in the study. Recruitment volume is capped at a maximum of 10 new participants per day to ensure a manageable workload for intervention and research staff throughout the study period. Once 10 individuals are randomized, no new registered users are invited for the remainder of the 24-hour period. Recruitment began in March 2012. As of October 30, 2013, 3602 participants have been randomized.

We defined the New Year period based on a clear peak and drop in the number of individuals that registered on BecomeAnEX between December 1, 2012 and January 31, 2013. The average conversion rate of unique visitors to registrants each day from December 1 through December 25 was approximately 4.7%. This proportion increased almost 2-fold on December 26, 2012 to 8.2% and stayed elevated through January 9, 2013, at an average daily conversion rate of 7.4%. Thus, we selected this 15-day period as our New Year period. For comparison, we selected 2 other 15-day periods during the year based on several criteria: (1) similar marketing and promotion approach, (2) variations in season (ie, summer, fall), (3) same span of days of the week (Wednesday to Wednesday), and (4) roughly similar number of participants randomized during the designated time period. Based on these factors, 2 separate 15-day periods were selected for comparison: 1 during the summer (July 18, 2012 to August 1, 2012) and 1 during the fall (November 7, 2012 to November 21, 2012). We deliberately selected the fall period to include another popular quitting holiday, the American Cancer Society’s Great American Smokeout, which falls on the third Thursday of November (November 15, 2012). Inclusion of this time frame enabled us to compare participants enrolling during the New Year to participants potentially enrolling in response to another seasonal trigger for cessation.

### Interventions

Participants in all 4 treatment groups had full access to the BecomeAnEX website which provides assistance setting a quit date, assessment of motivation and nicotine dependence, problem-solving/skills training to enhance self-efficacy for quitting, assistance in selecting and using US Food and Drug Administration (FDA)-approved pharmacotherapies, and social support through a large online community [14,15]. Participants randomized to receive the social network intervention received proactive communications from established members of the BecomeAnEX community (integrators). Within 24 hours after a new participant joined the study, the integrators posted a public message on the new member’s profile page to welcome them to the site, encourage them to fill out their profile, or comment on some aspect of an existing profile. Participants randomized to receive NRT products from the study were mailed a free 4-week supply of the NRT product of their choice (patch, gum, or lozenge) within 3 days of randomization. The NRT is provided as an over-the-counter product (ie, with no additional support or guidance provided) to parallel the experience participants would have if they purchased NRT on their own.

### Data Collection

Data are obtained through 3 sources: (1) a Web-based clinical trials management system that automated the recruitment and enrollment process, (2) self-report assessments at baseline, 3-, and 9-months postrandomization, and (3) online tracking software that records utilization of BecomeAnEX. Our analyses of smoking outcomes focus on the 3-month follow-up because this is typically when treatment utilization and intervention effects are the strongest. Telephone follow-up by professional telephone interviewers blinded to treatment condition for online nonresponders is used to maximize follow-up rates. Participants are reimbursed via Amazon or PayPal for survey completion (US $20 for Web survey, US $15 for phone survey). Individual level tracking metrics of BecomeAnEX utilization are recorded using Adobe/Omniture SiteCatalyst [16] software.

### Measures

#### Overview

The following measures from the parent trial were examined for these analyses.

#### Sociodemographic Variables

Participants reported age, gender, race, ethnicity, marital status, employment, education, and type of Internet connection.

#### Smoking Variables

At baseline, participants completed the Fagerström Test for Nicotine Dependence (FTND) [17] and also reported their confidence and desire to quit smoking (1=not at all, 5=very much), the current number of cigarettes smoked per day, the number of quit attempts in the previous year, and motivation to quit [18].

#### Psychosocial Variables

The appraisal and belonging subscales of the 12-item Interpersonal Support Evaluation List (ISEL) [19] were used to measure perceived availability of social resources at baseline. The appraisal subscale measures the perceived availability of someone to talk to about one’s problems; the belonging subscale assesses the perceived availability of people with whom to engage in activities. Perceptions of cessation-related social support are measured at baseline and follow-up with a 6-item version of the Partner Interaction Questionnaire [20,21] that assesses receipt of positive behaviors (supportive of cessation) and negative behaviors (harmful to cessation) from an individual who has followed the participant’s efforts to quit smoking.

#### Treatment Adherence

Website utilization during the first 3 months of the study was extracted from the BecomeAnEX database and included the following metrics: number of log-ins, minutes spent using the site during each visit/session, number of pages viewed during each visit/session, and the number of blog posts read and made. Website utilization was recorded using Adobe/Omniture SiteCatalyst. Each page view by a participant was recorded into a relational database, and page views were grouped into sessions. The duration of a session was defined as the time elapsed between the first page view and the last page view in a given session. If a user did not view a new page for more than 30 minutes, the system marked them as inactive and their next return visit created a new session. At each follow-up, participants reported use of NRT and prescription cessation medications (eg, Chantix, bupropion).

#### Three-Month Outcome Measures

Smoking outcomes examined in these analyses included self-reported point prevalence abstinence (30 day and 7 day) measured at 3 months. We also examined the number of quit attempts reported at 3 months.

### Statistical Analyses

The effects of recruitment phase (New Year, summer, fall) on recruitment metrics, baseline characteristics, treatment utilization, and outcome measures were evaluated via chi-square tests for proportions or 1-way ANOVA, depending on whether the metrics were proportions or continuous variables. Significant omnibus tests were followed by unadjusted pairwise comparisons. Analyses were conducted on the full sample of participants recruited in each phase (ie, collapsed across treatment groups).

## Results

### Recruitment and Retention by Recruitment Phase

Table 1 shows the Consolidated Standards of Reporting Trials (CONSORT) metrics of each recruitment phase. We examined advertising expenditures during the 2 weeks before each recruitment phase in addition to the recruitment phase itself. For the New Year, summer, and fall phases, expenditures totaled US $57,508, US $48,632, and US $38,374, respectively. The conversion rate of unique visitors to new registered users during the New Year period (7.4%) was significantly higher than both summer (4.6%) and fall (4.9%) periods; summer and fall conversion rates did not differ. Among new registered users during the New Year period, 868 were invited to participate in the study. Of these, 44.1% (383/868) accepted the invitation and completed eligibility screening; 67.1% (257/383) were eligible, 93.0% (239/257) consented, and 52.9% of those eligible (136/257) completed the baseline assessment and were randomized to treatment. Among the New Year participants, 58.8% (80/136) completed the 3-month follow-up survey. With the exception of conversion rate, there were no significant differences between recruitment phases noted for any of the CONSORT metrics.

### Baseline Characteristics by Recruitment Phase

New Year participants differed from participants recruited during other time periods on age, education, employment, and marital status (see Table 2). New Year participants were older (mean 43.2, SD 12.3 vs mean 39.1, SD 13.3; *P*=.01) and more likely to be employed full time (58.9%, 79/136 vs 40.6%, 43/106; *P*=.01) compared to summer participants. New Year participants were more likely to have attended college than both summer and fall participants (New Year: 80.9%, 110/136; summer: 66.0%, 70/106; fall: 68.7%, 68/99; *P*=.02). Both New Year and summer participants were more likely to have a spouse/partner compared to fall participants (New Year: 63.2%, 86/136; summer: 65.1%, 69/106; fall: 47.5%, 47/99; *P*=.02). Summer and fall participants differed on Internet access (*P*=.004), but were not different from New Year enrollees.

### Treatment Utilization Metrics by Recruitment Phase

Among the various utilization metrics we examined (Table 3), only the number of total Web pages viewed differed significantly between the 3 groups of participants: page views was higher among New Year participants (median 57, IQR 20-57) than both summer (median 29, IQR 13-59; *P*=.002) and fall enrollees (median 36, IQR 19-69; *P*=.004). Summer and fall participants did not differ on page views.

### Smoking Outcomes by Time of Enrollment

There were no significant differences in any of the smoking outcomes we examined by recruitment phase. Using intention-to-treat analyses, 30-day point prevalence abstinence was 11.8% (16/136), 15.1% (16/106), and 17.2% (17/99), and 7-day point prevalence abstinence was 16.2% (22/136), 18.9% (20/106), and 22.2% (22/99) for New Year, summer, and fall participants, respectively. Using responder-only analysis, 30-day point prevalence abstinence was 20% (16/79), 23% (16/68), and 32% (17/53), and 7-day point prevalence abstinence was 28% (22/79), 29% (20/68), and 41% (22/53) for New Year, summer, and fall participants, respectively. There was no difference in the number of quit attempts reported by the 3 groups at the 3-month follow-up (Table 4).

**Table 1:** Recruitment and retention metrics by recruitment phase.

Recruitment and retention metrics	New Year	Summer	Fall	*P* value
Total advertising expenditure (US $)	57,508	48,632	38,374	—
Unique visitors, n	32,853	30,605	22,017	—
New registered users, n	2424	1404	1079	—
Conversion rate, %	7.4	4.6	4.9	.001
Invited to study, n	868	792	594	—
Accepted invitation, n (% of invited to study)	383 (44.1)	325 (41.0)	270 (45.5)	.21
Eligible, n (% of accepted invitation)	257 (67.1)	218 (67.1)	186 (68.9)	.88
Consented, n (% of eligible)	239 (93.0)	205 (94.0)	176 (94.6)	.77
Randomized, n (% of eligible)	136 (52.9)	106 (48.6)	99 (53.2)	.79
Completed 3-month follow-up, n (% randomized)	80 (58.8)	69 (65.1)	54 (54.5)	.30

**Table 2:** Baseline characteristics by recruitment phase.

Baseline characteristics			New Year n=136	Summer n=106	Fall n=99	*P* value
**Demographic variables**						
	Age (years), mean (SD)		43.2 (12.3)	39.1 (13.3)	40.6 (13.1)	.02
	Sex (female), n (%)		97 (71.3)	76 (71.7)	68 (68.7)	.87
	**Race, n (%)**					.78
		Non-white	22 (16.2)	14 (13.2)	16 (16.2)	
		White	114 (83.8)	92 (86.8)	83 (83.8)	
	Ethnicity (Hispanic), n (%)		8 (5.9)	3 (2.8)	3 (3.0)	.45
	**Education, n (%)**					.02
		High school or less	26 (19.1)	36 (34.0)	31 (31.3)	
		Some college or more	110 (80.9)	70 (66.0)	68 (68.7)	
	**Employment status, n (%)**					.02
		Full time	79 (58.9)	43 (40.6)	52 (52.5)	
		Not full time	57 (41.9)	63 (59.4)	47 (47.4)	
	**Marital status, n (%)**					.02
		Partner	86 (63.2)	69 (65.1)	47 (47.5)	
		No partner	50 (36.8)	37 (34.9)	52 (52.5)	
**Smoking variables**						
	Cigarettes per day, mean (SD)		17.3 (8.1)	16.4 (7.6)	16.2 (8.3)	.54
	**Motivation to quit,** ^a^ ** n (%)**					.58
		Next 30 days	116 (85.9)	87 (82.1)	86 (87.9)	
		Next 6 months	19 (14.1)	19 (17.9)	13 (13.1)	
	Desire to quit, mean (SD)		4.6 (0.6)	4.6 (0.6)	4.6 (0.6)	.94
	Confidence in quitting, mean (SD)		3.3 (1.0)	3.3 (1.0)	3.4 (1.2)	.81
	Quit attempts past year, mean (SD)		2.4 (3.0)	1.8 (2.6)	2.6 (3.3)	.10
	FTND,^b^ mean (SD)		5.6 (2.2)	5.2 (2.2)	5.0 (2.1)	.08
**Psychosocial variables**						
	**Partner Interaction Questionnaire, mean (SD)**					
		Positive subscale	9.1 (2.9)	9.8 (2.3)	9.3 (3.0)	.52
		Negative subscale	6.8 (4.2)	5.9 (4.1)	7.1 (4.0)	.25
	**ISEL,** ^c^ ** mean (SD)**					
		Appraisal subscale	8.2 (3.4)	8.1 (3.1)	8.4 (3.3)	.77
		Belonging subscale	7.8 (2.9)	7.9 (3.1)	8.4 (3.0)	.29
	**Internet access,** ^d^ ** n (%)**					.02
		High speed/broadband	108 (80.0)	92 (88.5)	72 (72.7)	
		Mobile device	27 (20.0)	12 (11.5)	27 (27.3)	

^a^Motivation to quit excluded 1 participant who reported no plans to quit smoking in New Year group.

^b^FTND: Fagerström Test for Nicotine Dependence.

^c^ISEL: Interpersonal Support Evaluation List.

^d^Internet access: n=3 cases dropped that reported using a dial-up connection (summer: n=2; New Year: n=1; fall: n=0).

**Table 3:** Treatment utilization metrics by recruitment phase.

Treatment utilization metrics		New Year n=136	Summer n=106	Fall n=99	*P* value
Log-ins, median (IQR)^a^		2 (1-4)	2 (1-4)	2 (1-5)	.63
**Return visits, n (%)**					.73
	None	50 (36.8)	48 (45.3)	42 (42.4)	
	1	28 (20.6)	18 (17.0)	17 (17.2)	
	≥2	58 (42.6)	40 (37.7)	40 (40.4)	
Time on site, median (IQR)		41 (20.5-86)	29 (13-59)	40 (16.5-64)	.11
Page views, median (IQR)		57 (20-57)	29 (13-59)	36 (19-69)	.02
**Blogs read, n (%)**					.71
	None	95 (69.9)	72 (67.9)	71 (71.7)	
	1	9 (6.6)	12 (11.3)	9 (9.1)	
	≥2	32 (23.5)	22 (20.8)	19 (19.2)	
**Blog posts, n (%)**					.45
	None	117 (86.0)	95 (89.6)	89 (89.9)	
	1	3 (2.2)	4 (3.8)	4 (4.0)	
	≥2	16 (11.8)	7 (6.6)	6 (6.1)	
Any NRT use (yes), n (%)		48 (60.0)	37 (53.6)	36 (66.7)	.34

^a^12 missing values (New Year: n=7; summer: n=3; fall: n=2).

**Table 4:** Smoking outcomes by recruitment phase.

Smoking outcomes		New Year	Summer	Fall	*P* value
**30-day point prevalence abstinence, n (%)**					
	Intention-to-treat	16 (11.8)	16 (15.1)	17 (17.2)	.33
	Responder only	16 (20.0)	16 (23.2)	17 (31.5)	.31
**7-day point prevalence abstinence, n (%)**					
	Intention-to-treat	22 (16.2)	20 (18.9)	22 (22.2)	.27
	Responder only	22 (27.5)	20 (29.0)	22 (40.7)	.23
Quit attempts, mean (SD)		3.3 (4.1)	4.3 (8.6)	3.7 (4.7)	.58

## Discussion

### Principal Findings

The results of this study indicate that smokers visiting a Web-based cessation program during the New Year period are more likely to register for treatment and differ on several demographic variables, but show similar patterns of treatment engagement, retention, and short-term cessation outcomes compared with participants who visit the site during other periods of the year. Our hypotheses that New Year participants would differ on measures of motivation and desire to quit were not supported, and there were no differences on any of the smoking variables we examined. In addition, our hypotheses about lower retention rates, website utilization rates, and cessation outcomes were also not supported. Follow-up rates were comparable across all 3 periods, and smokers recruited during the New Year period quit at the same rate as smokers recruited at other times during the year. These results mitigate scientific concerns about recruiting participants during this time frame and are reassuring for researchers conducting Web-based cessation trials.

Our findings that New Year participants were older, more educated, more likely to be employed full time, and more likely to have a relationship partner may suggest that smokers with greater resources are more affected by the seasonal trends of quitting around the New Year. Alternatively, these differences may be a function of differential message exposure: older employed individuals may have been more likely to be impacted by the BecomeAnEX online advertising campaign, or reminded of cessation through workplace wellness programs or other promotional activities. Although not significant, smokers recruited during the New Year period also had a higher level of nicotine dependence and a higher number of previous quit attempts at baseline, also suggesting that seasonal trends may serve as a cue to action for more dependent and motivated smokers. We do not have an explanation for the finding that New Year participants viewed more website pages than participants in other recruitment phases did, especially because no other metric of engagement or utilization was significantly different.

To our knowledge, this is the first study to examine demographic, utilization, and outcome patterns of smokers recruited to a Web-based intervention during the New Year period, although there is interest in seasonal patterns of behavior. Delnevo and colleagues [10] noted seasonal patterns in motivation for quitting among callers to a quitline and discussed the implications for planning, promoting, and evaluating telephone quitlines. Using Internet search query data, Ayers et al [22] documented seasonality in searches for mental health information, with increases in information seeking that corresponded to patterns of seasonal affective disorder. The lack of previous publications in this area may be because dramatic increases in recruitment are to some degree unique to the online environment and Web-based studies that are capable of enrolling a large number of trial participants in a relatively short period of time.

Our findings add to the small but growing literature on recruitment methods for Web-based tobacco interventions [23-31]. Most studies to date have focused on comparisons between online recruitment methods (eg, online banner ads, search engine advertising) and more traditional recruitment methods (eg, newspaper ads, targeted mailings) [24,25,32], evaluation of different Internet-based methods [28,29], or the use of offline methods (eg, physician referral) to drive tobacco users to Web-based interventions [30,31]. The primary endpoints of interest in most studies are baseline participant characteristics and recruitment yield and/or efficiency. Heffner et al [25] evaluated the impact of Web-based and traditional recruitment methods on 3-month data retention and 30-day point prevalence smoking abstinence at the 3-month outcome assessment in a cessation trial and found no differences by recruitment method. Our findings regarding the impact of seasonality are consistent with previous studies that have demonstrated some differences in the types of participants recruited to Web-based tobacco interventions, but no differences in their participation in or outcomes from such trials.

### Limitations

Several limitations to this study should be noted. We examined variations in participant characteristics for a single cessation website as part of an ongoing randomized trial. This site exists in a larger ecosystem of promotion, advertising, and branding as part of the national BecomeAnEX campaign, which has been in existence since 2008. Our results are likely related to the specific strategy employed by BecomeAnEX, which is largely online advertising in its present implementation. Other advertising and promotional strategies of different Web-based interventions could yield different results. In addition, our automated titration of recruitment volume should be noted when considering the pragmatic implications of our results. Although the number of visitors to BecomeAnEX was higher during the New Year period and a higher proportion of them registered to become members, the number of participants recruited to our clinical trial during different periods throughout the year has remained relatively constant because of the daily cap we have on enrollment. This cap is designed to maintain a consistent volume of participants for our research and intervention staff to manage. If this cap were not in place, we may have seen a higher number of participants invited to the study and differences in the proportion of participants accepting or declining the study invitation. This may have important pragmatic considerations for other Web-based trials that have human involvement, but we believe this is unlikely to have affected the other metrics we examined (ie, follow-up rates, cessation outcomes). The daily cap on recruitment may also have affected statistical power. The response rate to the 3-month follow-up is lower than desired despite numerous online and offline strategies to reach participants, but is comparable to or higher than other Internet studies [33].

### Conclusions

Internet interventions for health behavior change are characterized by their ability to recruit broadly and provide treatment at scale. Secular or temporal variations, such as the New Year holiday, and the associated media attention to smoking cessation and resolution making can result in large-scale swings in the number of individuals arriving at Web-based cessation interventions. For interventions that can effectively capture and enroll those individuals, seasonal variations could dramatically increase recruitment efficiency for clinical trials.

## Abbreviations

CONSORT: Consolidated Standards of Reporting Trials
FDA: US Food and Drug Administration
FTND: Fagerström Test for Nicotine Dependence
ISEL: Interpersonal Support Evaluation List
NRT: nicotine replacement therapy
